# Visualizing phylogenetic tree landscapes

**DOI:** 10.1186/s12859-017-1479-1

**Published:** 2017-02-02

**Authors:** James C. Wilgenbusch, Wen Huang, Kyle A. Gallivan

**Affiliations:** 1 Department of Scientific Computing, Florida State University, Tallahassee, FL 32306 USA; 2 Present Address: Minnesota Supercomputing Center, University of Minnesota, Minneapolis, 55455 USA; 3 Department of Mathematics, Florida State University, Tallahassee, FL 32306 USA; 4 Present Address: Department of Computational and Applied Mathematics, Rice University, Houston, TX 77005 USA

**Keywords:** Mitochondrial DNA, MDS, NLDR, Combining data, Visualization, Tree landscape, Bootstrap

## Abstract

**Background:**

Genomic-scale sequence alignments are increasingly used to infer phylogenies in order to better understand the processes and patterns of evolution. Different partitions within these new alignments (e.g., genes, codon positions, and structural features) often favor hundreds if not thousands of competing phylogenies. Summarizing and comparing phylogenies obtained from multi-source data sets using current consensus tree methods discards valuable information and can disguise potential methodological problems. Discovery of efficient and accurate dimensionality reduction methods used to display at once in 2- or 3- dimensions the relationship among these competing phylogenies will help practitioners diagnose the limits of current evolutionary models and potential problems with phylogenetic reconstruction methods when analyzing large multi-source data sets. We introduce several dimensionality reduction methods to visualize in 2- and 3-dimensions the relationship among competing phylogenies obtained from gene partitions found in three mid- to large-size mitochondrial genome alignments. We test the performance of these dimensionality reduction methods by applying several goodness-of-fit measures. The intrinsic dimensionality of each data set is also estimated to determine whether projections in 2- and 3-dimensions can be expected to reveal meaningful relationships among trees from different data partitions. Several new approaches to aid in the comparison of different phylogenetic landscapes are presented.

**Results:**

Curvilinear Components Analysis (CCA) and a stochastic gradient decent (SGD) optimization method give the best representation of the original tree-to-tree distance matrix for each of the three- mitochondrial genome alignments and greatly outperformed the method currently used to visualize tree landscapes. The CCA + SGD method converged at least as fast as previously applied methods for visualizing tree landscapes. We demonstrate for all three mtDNA alignments that 3D projections significantly increase the fit between the tree-to-tree distances and can facilitate the interpretation of the relationship among phylogenetic trees.

**Conclusions:**

We demonstrate that the choice of dimensionality reduction method can significantly influence the spatial relationship among a large set of competing phylogenetic trees. We highlight the importance of selecting a dimensionality reduction method to visualize large multi-locus phylogenetic landscapes and demonstrate that 3D projections of mitochondrial tree landscapes better capture the relationship among the trees being compared.

**Electronic supplementary material:**

The online version of this article (doi:10.1186/s12859-017-1479-1) contains supplementary material, which is available to authorized users.

## Background

The rapid increase in the availability of genomic-scale multiple sequence alignments covering diverse sets of taxa offers new and exciting opportunities for those seeking to understand the processes and patterns of molecular evolution and brings us a step closer to solving such grand challenges as assembling a Tree of Life. In practice however, regions (e.g., genes, codons, and structural features) of large multi-source data sets seldom support a single phylogenetic tree. More often than not, we are left to sort through hundreds if not thousands of competing phylogenies. Different data partitions may support different phylogenies because reconstruction methods sometimes fail to adequately accommodate process heterogeneous underlying data partitions found within an alignment [1–4] or because some data partitions simply do not share the same evolutionary history, (see Maddison [5] and references cited therein). Furthermore, large data sets are typically more computationally challenging to analyze and often call for more extreme heuristic shortcuts, which may fail to converge to a global optimum [6]. Therefore, visually representing the similarity or dissimilarity among competing phylogenic trees supported by different genes or by other a priori defined data partitions in 2 or 3-dimensional space is a potentially powerful way for investigators to gain a better perspective on the problems sometimes associated with the analysis of large multi-source data sets [7].

To date, the typical approach used to summarize a set of phylogenetic trees is to create a single consensus tree from the set of competing trees, in which the vertices of the consensus tree are only retained if they are shared by a majority of the trees contained within the set of candidate trees. Phylogenetic network [8] and maximum agreement subtree [9] methods also result in concise summaries for sets of conflicting trees whether the conflicts are caused by reticulate events or by modeling errors. These methods, while easy to interpret, lack information regarding the distribution and relationship among the candidate trees. Refinements to the consensus tree approach have been made by applying clustering methods to identify subsets of related phylogenies contained within the larger set [10]. An appealing aspect of this method is that it can be used as an objective means to identify discontinuities in the distribution of candidate phylogenetic trees or the phylogenetic landscape. However, the clustering approach still discards a great deal of information and lacks the fine-grain perspective needed to infer the cause of the discordance among the competing trees.

Motivated by the inherent limitations of the consensus tree approach, Amenta and Klinger [11] applied a dimensionality reduction method that they referred to as “iterative Multidimensional Scaling (MDS)” to display tree-to-tree distances in a 2-dimensional space. The practice of visually representing sets of competing phylogenetic trees in a geometric space can be separated into three major and sometimes computationally intensive components: 1) the selection of a set of phylogenic trees to be compared; 2) the calculation of pairwise distances between all members of the set of selected phylogenetic trees; and 3) the calculation of coordinates in 2 or 3-dimensional space, such that the Euclidean distance between the projected points closely corresponds to the original tree-to-tree distances. Hillis et al. [7] later applied the method developed by Amenta and Klinger [11] to demonstrate how this approach could be used to explore tree islands, compare trees from different data partitions, compare trees from bootstrap samples with trees sampled from a Markov Chain Monte Carlo (MCMC) simulation, and compare trees from different MCMC simulations.

While the aforementioned authors did an excellent job demonstrating the utility of this new approach, they did not specifically address some key methodological questions specifically related to mapping high-dimensional data, in this case tree-to-tree distance, into a lower dimension for visualization. In this study we specifically address some of these unanswered questions by applying several Nonlinear Dimensionality Reduction (NLDR) [12] methods to the problem of visualizing large sets of phylogenetic trees obtained from the analysis of whole mitochondrial DNA (mtDNA) genomes. The performance of these methods is evaluated relative to others and to the method introduced by Amenta and Klinger [11].

In addition, we estimate the intrinsic dimensionality of large collections of mtDNA gene trees in order to better understand whether viewing the relationship among phylogenetic trees in 3D is warranted. Finally, we introduce ways to compare projections obtained from unrelated alignments so that we might better understand the biological processes and methodological biases associated with the inference of phylogenetic trees. Correctly characterizing phylogenetic tree-space by dimensionality reduction methods is critical if this approach is to be of value as an interpretive or a diagnostic tool for detecting problems with substitution models or tree searching strategies.

## Methods

### Genomic data and phylogenetic analyses

Aligned whole mitochondrial DNA (mtDNA) genomes were obtained from three published studies representing Fishes (90 sequences) [13], Mammals (89 sequences) [14], and Salamanders (42 sequences) [15]. The software package PAUP* 4.0b10 [16] was used to perform 100-replicate nonparametric bootstrap analyses [17] on each of 15-gene partitions contained within each of the three-mtDNA alignments. Hereafter we will refer to the nonparametric bootstrap analyses as the bootstrap analyses. The maximum likelihood (ML) criterion and a heuristic search [neighbor joining starting tree, Sub-tree Pruning and Regrafting (SPR) branch swapping with a reconstruction limit of 10] were used to select optimal phylogenetic trees for each bootstrap replicate. Parameters of the ML model (i.e., nucleotide substitution rates, base frequencies [18], and an among site rate heterogeneity parameter [19]) were independently optimized for each gene partition on a neighbor joining tree constructed for each gene partition. A special purpose script [20] was used to distribute phylogenetic analyses in parallel on FSU’s shared HPC system.

Phylogenetic analyses as described above were also performed on a test data set consisting of 15 partitions equal in size to the original gene partitions found in the Salamander data set. The 15 test partitions were composed of characters (i.e., columns in the multiple sequence alignment) selected at random but in proportion to their occurrence in the original Salamander mtDNA genome alignment. Therefore, the partitions within the newly compiled test data set only differ from one another by sampling error and by size (i.e., number of columns). The test data set was intended to serve as a null data set in which any patterns observed in subsequent NLDR projections could only have resulted from sampling error and the size of data partitions.

### Tree-to-tree distances

A set of trees was compiled for each of the three multiple sequence alignments by taking the union of the bootstrap trees obtained from the analyses of the 15 individual gene partitions (Table 1). The Robinson-Foulds [21, 22] distance (RF-distance), as implemented in the software package PAUP* 4.0b10 [16], was used to measure the topological difference between all of the trees in each of the three concatenated sets of trees. The RF-distance counts the number of bipartitions that are present in one but not both trees being compared and is a commonly used tree-to-tree distance metric. We also calculated the geodesic distance [23] for each of the three-mtDNA data sets to determine whether the underlying distance metric had an impact on the results related to the NDLR methods or the dimensionality estimates implemented in this study. The geodesic is sometimes preferred because this distance naturally incorporates both the tree topology and branch (edge) lengths. Only the results using the RF distance are presented here because choice of distance did not alter our conclusions. The resulting RF distance matrices were used for all subsequent NLDR analyses described below.

**Table 1:** Characters per gene partition for each mtDNA data set

Gene	Fishes	Mammals	Salamanders	Test
ATP8	939(156)	362(164)	783(162)	768
ND4L	1362(285)	1056(290)	378(279)	271
ND3	690(339)	1559(347)	355(330)	236
COII	444(690)	433(682)	196(681)	121
ATP6	415(657)	540(708)	156(681)	111
COIII	643(783)	554(786)	149(783)	114
12S	256(693)	219(787)	119(809)	107
ND1	507(933)	170(969)	111(957)	107
ND2	371(990)	129(1048)	111(1014)	105
CytB	235(1164)	195(11 s40)	122(1131)	107
16S	205(922)	146(1199)	106(1260)	103
tRNAs	162(1152)	146(1339)	108(1274)	101
ND4	219(1371)	150(1384)	108(1332)	104
COI	386(1539)	228(1542)	106(1548)	102
ND5	188(1632)	114(1801)	103(1734)	102
Total Trees	7022(13,306)	6001(14,186)	3011(13,975)	2559

The number of unique ML bootstrap topologies (100 replicates, GTR + Γ) retained for each of the 15-mtDNA gene partitions for each of the three- mtDNA alignments. The number of nucleotides representing each gene partition is given in parentheses. Gene partitions are sorted in ascending order of their size. Characters for each test partition are selected at random but in proportion to the size of each partition from the original salamander alignment

### Intrinsic dimensionality measures

The utility of phylogenetic landscapes generated by dimensionality reduction methods depends on whether there exists a reliable representation of the tree-to-tree RF-distances in 2 or 3-dimensional space. For example, [7] demonstrated a simple case where the projection of 3D data into a 2D space is distorted in such a way that the original relationship among data is lost and subsequent interpretations of the 2D projection will be misleading. This result could also be obtained for higher dimensional data projected into either a 2D or 3D space. Whether the RF-distances for the three-mtDNA data sets used in this study suffer from the “curse of dimensionality” can, to a limited extent, be evaluated by estimating the intrinsic dimensionality of the tree-to-tree RF-distances. The intrinsic dimensionality of a data set can be thought of as a measure of the number of variables required to represent the original distances [12].

If the intrinsic dimensionality of the RF-distances is three then we should be able to represent these data in a 3D space with very little to no distortion. Alternatively, if the intrinsic dimensionality of the distances is greater than three then we will necessarily have to discard some information in order to visualize the data set. Whether the discarded information results in distortions that mislead our interpretation cannot be fully answered, except perhaps by a subjective evaluation of the projection. We employed four different methods to estimate the intrinsic dimensionality of the RF-distances using the Treescaper software package [24]; Correlation Dimension [25, 26], a maximum likelihood estimator [27], a Nearest Neighbor estimator [28] and by examining the final value of the NLDR cost function versus the dimension to which the data was reduced [12]. These methods are described in the Additional file 1.

### Methods of dimension reduction and evaluation

The NLDR methods evaluated in this study consist of two major components; 1) a stress function, which is an objective function used to evaluate embeddings of the RF-distances in lower dimensions, and 2) an algorithm used to optimize the stress function. The stress functions are Normalized stress [29], Kruskal-1 stress [30], Sammon’s stress, also known as the nonlinear mapping (NLM) stress [31], and Curvilinear Components Analysis (CCA) stress [32]. The optimization algorithms are majorization, Gauss-Seidel-Newton, stochastic gradient descent, and MCMC simulated annealing. To better understand how each of these constituent components contributes to the overall performance of the NLDR method we implemented in the software package Treescaper [24] 14 of the 16 possible combinations of the four-optimization algorithms and four stress functions for reasons discussed in the Additional file 1: Appendix.

When implementing each pairing of stress function and optimization algorithm, efficiencies specific to the pair were exploited. The details of stress functions, optimization algorithms, and implementation considerations are presented in the Additional file 1: Appendix. The values obtained by different stress functions cannot usefully be compared directly, therefore several goodness of fit measures were used to evaluate how well each of the four stress functions were at characterizing the original RF-distances. They are 1 Nearest Neighbor (1NN) [33], Continuity [34], and Trustworthiness [34]. Details of each goodness of fit measure are presented in the Additional file 1: Appendix.

## Results and discussion

### Phylogenetic analyses

The number of bootstrap trees (i.e., the “raw data” of our subsequent analyses) representing each gene partition varied from between 103 and 1559 (Table 1). In general, shorter gene partitions (i.e., partitions with fewer nucleotide characters per sequence) are represented by more bootstrap trees [35, 36]. The inverse relationship between gene partition length and number of bootstrap trees suggests that shorter gene partitions do not have a sufficient number of informative characters for the analyses to discriminate among competing tree topologies.

**Fig. 1:**
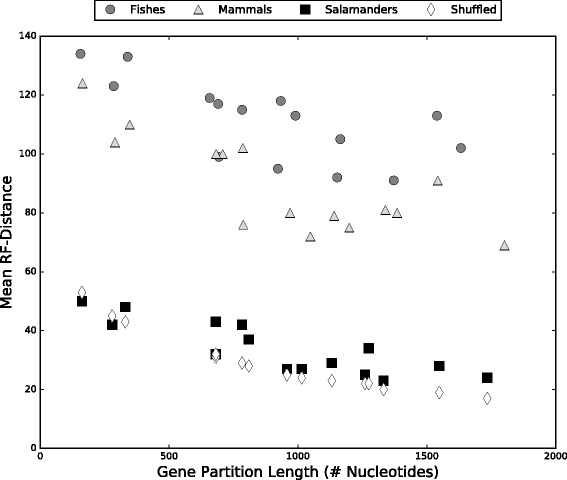
Mean RF-distances by mtDNA gene. Mean RF-distances plotted against the length (number of nucleotides) of each gene partition for each set of taxa

**Fig. 2:**
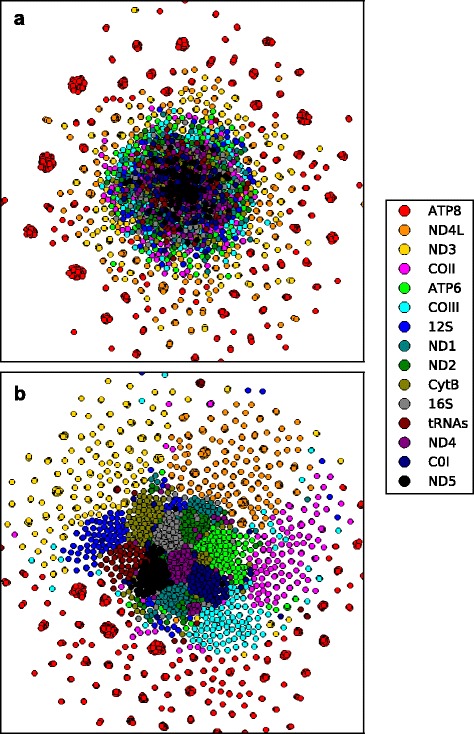
Phylogenetic landscape of shuffled versus unshuffled mtDNA data. Projections of the RF-distances among bootstrap trees from separate analyses of 15 mtDNA data partitions from within the (**a**) the test data set generated by shuffling columns in the original Salamander alignment and (**b**) the original salamander mtDNA alignment [15]. Colors correspond to the bootstrap trees found by each separate data partition analysis

Not only are more trees retained by bootstrap analyses of shorter gene partitions, but the mean RF-distance among trees from shorter partitions is generally greater then the mean RF-distances among bootstrap trees from longer gene partitions (Fig. 1). This relationship was also observed in the test (“Shuffled”) data set (Fig. 1), where partitions equal in size to those in the original salamander alignment were created by selecting characters at random from the entire salamander genome. By homogenizing the mtDNA characters from the 15 gene partitions over a range of partition lengths we were able to evaluate how partition length influences mean RF-distance. The relationship between partition length and the number and distance among bootstrap trees is germane to this work because it begins to shape what we might expect to observe when the RF-distances are plotted as tree landscapes using NLDR methods. For example, based solely on the length of a data partition, we will expect to see a greater number of more widely distributed trees from the bootstrap analyses of smaller gene partitions (Fig. 2a). Additional structure or patterns in the NDLR plots will either be attributed to our nucleotide substitution models failing to accommodate the underlying process heterogeneity associated with each of the 15 gene partitions or our tree searching methods systematically failing to converge.

The bootstrap analysis, as applied in phylogenetics, uses randomly selected columns from the original multiple sequence alignment to generate new character matrices, which are then used to infer optimal tree topologies [17]. If all, or most of the characters contained within a given gene partition support the same phylogeny, then each bootstrap replicate data set will unambiguously support a single tree topology. This level of congruence rarely occurs in real data sets, however. Bootstrap analyses typically result in a group of related trees, which in effect represent a confidence interval around the evolutionary history of that gene partition [2, 37]. By concatenating all trees from each of the separate bootstrap analyses, calculating their pairwise RF-distance, and projecting these distances in 2 or 3D space we can at once see the relationship among the trees from the separate gene partitions [7]. If our nucleotide substitution models and tree searching methods worked perfectly then the bootstrap trees from each mtDNA gene partition would mostly overlap because the gene partitions are physically linked on the mtDNA genome and therefore must share a common history. It is generally accepted that our models and methods are not perfect [6]; therefore, it should come as no surprise that trees from within a given bootstrap analysis are more similar to one another than are trees from different bootstrap analyses and a NLDR method should clearly show these clusters of related gene trees (Fig. 2b). Failure of an NLDR method to show clusters of related trees could mislead practitioners to believe that their choice of tree reconstruction method correctly compensated for process heterogeneity. The ability of the NLDR methods to preserve these sub-clusters of related RF-distances contained within the concatenated bootstrap trees will, in large part, be used as the means by which we visually evaluate the success of an NDLR method.

### Intrinsic dimensionality of tree-to-tree distances

**Fig. 3:**
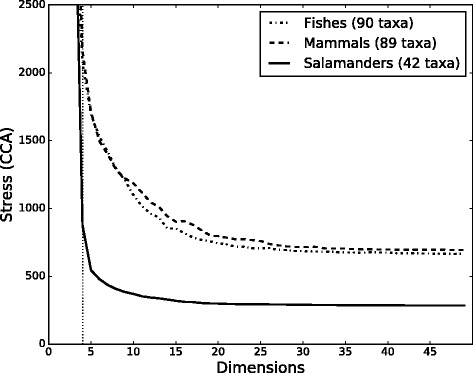
Dimensionality of tree space. The CCA stress plotted against the projection dimensionality for each of the three mtDNA alignments

We used four different methods to estimate the intrinsic dimensionality of each of the three tree-to-tree distance matrices generated from the concatenated bootstrap analyses. Our estimates of the intrinsic dimensionality for each data set varied from between 3 to 15-dimensions (Table 2). These estimates show that the use of 3D projections is warranted for viewing the mtDNA tree landscapes in order to minimize loss of information and to preserve the relationship among bootstrap trees suggested by the RF-distances. Plotting the CCA stress as a function of dimensions shows that using more than 15 dimensions does very little to improve the fit of the projected distances with those obtained using the RF-distance metric (Fig. 3). While most of the estimates of intrinsic dimensionality suggest that viewing the distances in 2 and 3D will result in some distortion of the relationship among the RF-distances, it is less obvious as to whether this distortion can impact our interpretation of how the trees are related. For example, 2 and 3 dimensions may adequately characterize the relative positions of clusters of gene trees to one another, while perhaps failing to more completely capture the relationship among the trees within each sub-cluster. Furthermore, other methodological considerations may be of as much or greater significance concerning the preservation of the original RF-distances. For example, we will demonstrate in the next section, that the choice of NLDR method can also significantly influence how trees are displayed in 2 and 3D and the choice of NLDR method may do more to distort or obscure the true relationship among large sets of trees than the number of dimensions into which they are projected.

**Table 2:** Dimensionality of tree-to-tree distance matrices

Measure	Fishes	Mammals	Salamanders
NN	3.37	3.41	3.94
COR	14.35	11.77	5.27
ML	6.61	6.21	7.33
Visual Inspection	15	15	7

The intrinsic dimensionality of each tree-to-tree distance matrices, where *NN* Nearest Neighbor estimator [28], *COR* Correlation Dimension [25, 26], *ML* Maximum Likelihood estimator [27], and “Visual Inspection” is based on results from Fig. 3 [12]

### Nonlinear dimensionality reduction

In order to better understand the effect of each constituent component of the NLDR analysis, the results from 14 of the 16 combinations of cost functions and optimization algorithms were evaluated. The combination of Majorization with CCA and Gauss-Seidel with CCA were not included for the reasons discussed in the Additional file 1: Appendix. The combination of SGD with the Kruskal-1 stress function was replaced by Kruskal-1 with a fixed-step classical deterministic steepest descent iteration, i.e., the step was not chosen to guarantee a true descent step, for the reasons also discussed in the Additional file 1: Appendix. This iteration is called Linear Iteration in [11] and will be so-called in the following discussions and figures. Each combination of cost function and optimization algorithm was run 10 times for each data sets using a different set of initial conditions. Like phylogenetic tree searching, NLDR is non-convex. By including results obtained from multiple starting points we are able to measure how results vary from one iteration of the same analysis to the next. We report the means and standard errors calculated using all ten iterations. Lastly, and perhaps most importantly, we visually compare the resulting projections to understand the extent to which the different NLDR methods influence our interpretation of the tree landscapes. For example, if a projection is deemed a better representation of the RF-distances by one or more of our objective measures, we want to know if it is possible to visually discriminate among the projections.

**Fig. 4:**
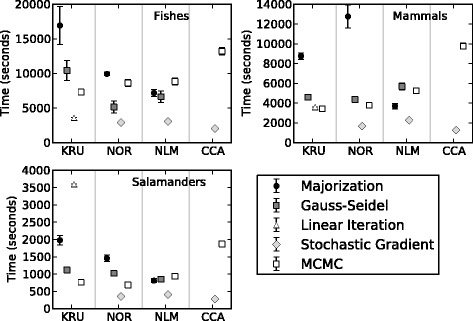
Time to Convergence. The average time in seconds it took for each combination of cost function and optimization algorithm used for the NLDR analysis of the three mtDNA RF-distance matrices to reach convergence. Each graph showing the results for the three mtDNA data sets is divided into four panes representing four cost functions (KRU = Kruskal-1; NOR = Normalized; NLM = Nonlinear Mapping; and CCA = Curverlinear Components Analysis)

All of the NLDR analyses that we evaluated took between four and 230 min to converge on local minima (Fig. 4). Our results show that on average the SGD algorithm converges faster than did the other optimization algorithms over all of the cost functions and each of the three mtDNA data sets. There is no clear trend among the three data sets as to which of four optimization algorithms converged most rapidly for the Kruskal-1 cost function. From a practical standpoint, these results are encouraging because they suggests that large data can be projected in 2- and 3D within a reasonable timeframe using the methods discussed herein. Furthermore, algorithmic refinements and some recently developed parallel NLDR implementations [38] promise to further improve run times and thereby will increase the practical potential of this general approach.

**Fig. 5:**
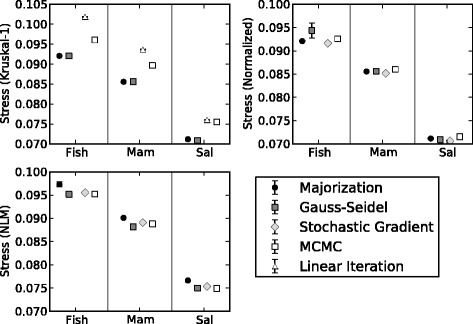
Minimizing the stress function. Raw stress values for three difference stress functions [Kruskal-1, Normalized, and Nonlinear Mapping (NLM)] were plotted for each data set and optimization algorithm

Time to convergence is only useful in light of how well each of the optimization algorithms is able to minimize their respective cost functions. For example, majorization took the longest time to minimize the Kruskal-1 cost function among all the other optimization algorithms that we compared (Fig. 4); however, majorization converged on a value that was as low or lower than most of the other optimization algorithms for each of the three data sets (Fig. 5). The Normalized and Nonlinear Mapping raw stress values are nearly identical for each of the optimization algorithms compared (Fig. 5) indicating that per unit time the SGD optimization algorithm is more efficient at minimizing these cost functions (Fig. 4). The raw stress for CCA was not plotted because this cost function contains a weighting function, which is used to preserve the relationship among local distances, but as a consequence makes it impossible to meaningfully compare the raw stress values from one CCA analysis to the next. Overall, SGD when used in conjunction with CCA converged faster than all other combinations of optimization algorithm and cost function (Fig. 4).

We evaluated the relative performance of each cost function using three measures of goodness of fit. These measures provide a common objective function that can be used to evaluate how well each projection preserves the relationship among the bootstrap trees as suggested by the original RF-distances. We chose to evaluate the projections with the lowest stress value for each cost function no matter which optimization algorithm was used to obtain it. In this way we restricted our comparisons to the best representation of the cost function given the optimization algorithms that were included in this analysis. According to both the 1-NN [33] and Trustworthiness [34] measures, the CCA cost function best preserves the relationship among all three of the original distance matrices. The CCA cost function also ranks highly among the cost functions preserving continuity, whereas Kruskal-1 ranks lowest among all three data sets. That CCA performs well according to all three measures of goodness of fit is not surprising. The RF-distance matrix contains groups or clusters of related distances that correspond to the bootstrap trees obtained from the independent analyses of 15 mtDNA genes. The flexible weighting function (F-lambda) included in the CCA method allows for tearing of the distance manifold [12] such that closely related RF-distances are drawn closer (i.e., continuity) without drawing more distant pairs closer (i.e., trustworthiness) to one another compared to how they are represented in the original distance matrix.

**Fig. 6:**
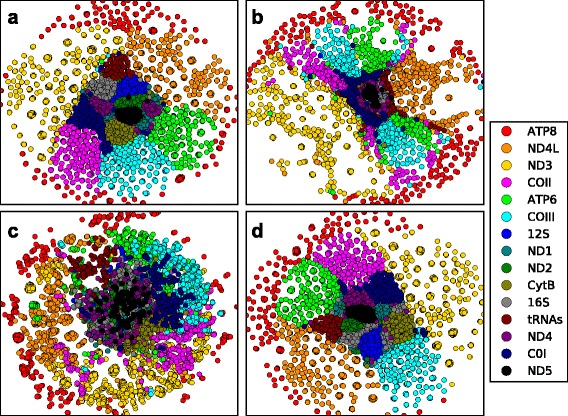
Phylogenetic tree landscape by NLDR method. Projections of RF-distances representing the concatenated bootstrap trees from 15 mammal mtDNA genes. Colors represent mtDNA bootstrap trees. Projections correspond to following cost function and optimization algorithm; **a** CCA plus SGD, **b** CCA plus MCMC, **c** NLM plus SGD, and **d** an alternative minima of CCA plus SGD

**Fig. 7:**
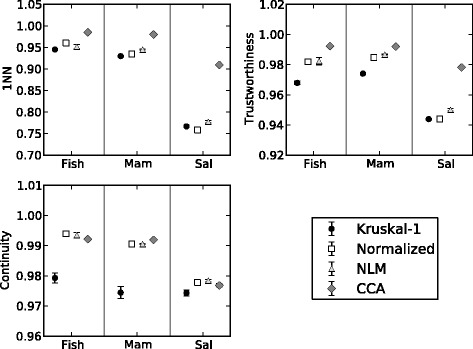
Goodness of fit. The mean goodness fit (with standard error bars) for ten initial conditions plotted for each cost function and data set

The choice of cost function and optimization algorithm used to project the RF-distances in 2 and 3D space significantly impacts the visual interpretation of the projected RF-distances. More importantly, it was not necessary to visually compare extreme cases to detect these differences. For example, Fig. 6a was created by using CCA plus SGD and represents the best projection of the mammal mtDNA bootstrap trees as judged by all three of the goodness of fit measures (Fig. 7). Changing the optimization algorithm for the CCA cost function to that which performs second best (MCMC) we see a loss of continuity among related groups in Fig. 6b for trees that are tightly grouped in plot A (e.g., see COIII trees). Furthermore, it is impossible to discriminated among some groups in Fig. 6b because many of the points are superimposed. Projections of the second best performing cost function and the SGD optimization algorithm (Fig. 6c), as judged by the goodness of fit measure, also gives a picture that lacks continuity when compared to Fig. 6a.

While the choice of optimization algorithm and cost function make a noticeable difference in the projection of mtDNA distances, different initial conditions for a given method are difficult to discern. For example, Fig. 6a and D represent two non-equivalent projections using the same cost function and optimization algorithm but a different set of initial conditions. We used the ordinary Procrustes analysis to characterize the dissimilarity among the ten projections obtained by using different initial conditions for a given combination of cost function and optimization algorithm. The Procrustes analysis leaves us with differences that have been adjusted for translation, rotation, and scale. Two projections are considered non-equivalent if the residual of Procrustes analysis is very large relative to the others. For the CCA plus SGD projections nine of the 10 projections were considered equivalent by this method and those that differed are shown as Fig. 6a and d. While Fig. 6a and d are considered to be non-equivalent by the Procrustes, the clusters of related gene trees within each plot are still well defined and also occupy similar positions with respect to one another especially when compared to the two other projects, wherein a different cost function (Fig. 6b) and a different optimization algorithm (Fig. 6c) were used.

### Comparing tree landscapes

**Fig. 8:**
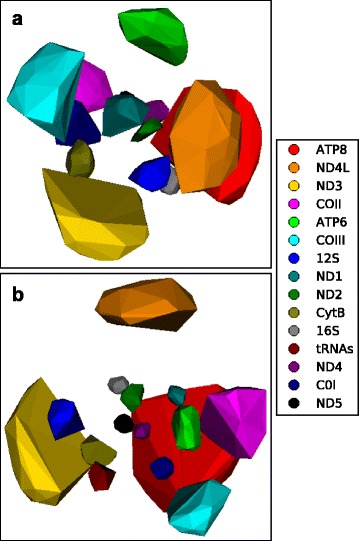
mtDNA genes in 3D convex hulls. The distribution of mtDNA gene trees for (**a**) Mammals and (**b**) Salamanders 3D convex hulls covering. Clusters of gene trees are represented by 3D convex hulls

**Fig. 9:**
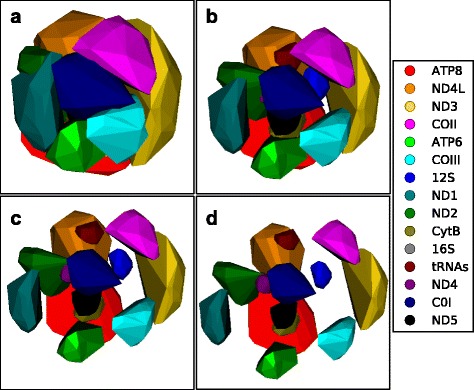
mtDNA genes in 3D convex hulls with seperation. 3D convex hulls cover points representing bootstrap trees obtained from separate analyses of the 15 mtDNA data partitions from within the Fishes mtDNA alignment. Plots were made by projecting RF-distances of the concatenated bootstrap trees using CCA plus SGD. Convex hulls in plots A through D were progressively moved away from the graph origin to reveal more of the clusters located near the graph origin

Hillis et al. [7] did a thorough job demonstrating a variety of applications for phylogenetic trees projected into a 2D space. In their exposition, they also briefly mentioned the idea of using ellipses to create 95% confidence intervals around projected trees obtained by bootstrap and Bayesian analyses. They did not implement this approach, however, citing potential interpretation problems related to mapping high-dimensional data into 2-dimensions and concerns about the statistical interpretation of these projections. We share their concerns, but also see the potential utility of an approach that attempts to visually relate a priori defined set of points in an NLDR projection. To this end, we implemented a method that encloses sets of points representing bootstrap trees from gene partitions in a convex hull. Visually grouping related trees in a convex hull can make it easier to interpret the significance of the size and relative position of clusters of trees in a single tree landscape and can also facilitate comparisons of multiple tree landscapes generated from different sets of taxa with similar data partitions (Fig. 8). In order to see “interior” clusters we devised a method for eliminating outlying trees from the set of points used to create the convex hull and for drawing clusters apart to reveal clusters located near to the graph origin (Fig. 9). A point was considered an outlier and removed from a set of points if the variance of the distance among all points decreased by an arbitrary threshold value (set to 0.01 in Fig. 8) when the point was excluded from the variance calculation. Changing the threshold value will determine how aggressively points are eliminated from a set of points. Convex hulls are moved away from the origin of the graph by translating each convex hull in a parallel manner in the direction from the center of all points to the center of the convex hull. Spreading convex hulls out in this way will change some relationships among the clusters within a single graph; however, different plots can still be compared usefully if the convex hulls are moved uniformly apart. This approach addresses the misgivings of [7] by displaying results in 3D to minimize distortion and by avoiding a strict statistical interpretation of the convex hulls.

## Conclusions

Correctly characterizing phylogenetic tree-space by dimensionality reduction methods is critical if this approach is to be of value as an interpretive or a diagnostic tool for large sets of trees obtained from whole genomes or from multi-gene data sets. We found that different dimensionality reduction methods can significantly influence the appearance, and hence interpretation of 2- and 3-D projections of tree-to-tree distances. In particular, among the cost functions and optimization algorithms that we evaluated, we found that CCA and the SGD method gave the best representation of the original tree-to-tree distances as indicated by the trustworthiness and continuity metrics. We also demonstrate by using several different estimates that the intrinsic dimensionality of three mtDNA tree-to-tree distance matrices is greater than two, and therefore using 3D to view these data is warranted in order to minimize distortions related to projecting high dimensionality data into a lower dimension. Tree landscapes obtained from analyses of alignments composed of very different sets of taxa but similar data partitions share some striking similarities. These similarities are easiest to observe when outliers are removed and related points are covered by a convex hull. The results obtained in this study establish that the choice of NLDR method can significantly influence our interpretation of tree landscapes. Perhaps more importantly, this work establishes the necessary framework for the application NLDR to be used in the evaluation of tree reconstruction methods, nucleotide substitution models, and other tree-to-tree distance matrices [e.g., Nearest Neighbor Interchange [39], Quartet [40], Subtree Prune and Regraft [41], Branch Score [42], Geodesic [43], Match [44].

## Additional file

Additional file 1: Appendix. Detailed description of the intrinsic dimensionality measures, nonlinear dimensionality reduction methods, and the goodness of fit measures [45–49]. (PDF 195 kb)

## Abbreviations

1NN: 1 Nearest Neighbor
2D: 2-dimensional
3D: 3-dimensional
CCA: Curvilinear Components Analysis
COIII: Mitochondrial cytochrome oxidase III
COR: Correlation Dimension
KRU: Kruskal-1
MCMC: Markov Chain Monte Carlo
MDS: Multidimensional Scaling
ML: Maximum likelihood
mtDNA: mitochondrial Deoxyribonucleic Acid
NLDR: Nonlinear Dimensionality Reduction
NLM: Nonlinear Mapping
NOR: Normalized
RF-distance: Robinson-foulds-distance
SGD: Stochastic Gradient Decent
SPR: Sub-tree Pruning and Regrafting

## References

[1] Alfaro ME, Huelsenbeck JP (2006). Comparative performance of Bayesian and AIC-based measures of phylogenetic model uncertainty. Syst Biol.

[2] Hillis D, Bull J (1993). An empirical test of bootstrapping as a method for assessing confidence in phylogenetic trees. Syst Biol.

[3] Nylander J, Ronquist F, Huelsenbeck J, Nieves-Aldrey J (2004). Bayesian phylogenetic analysis of combined data. Syst Biol.

[4] Pagel M, Meade A (2004). A phylogenetic mixture model for detecting pattern-heterogeneity in gene sequence or character-state data. Syst Biol.

[5] Maddison WP (1997). Gene trees in species trees. Syst Biol.

[6] Sanderson M, Kim J (2000). Parametric phylogenetics?. Syst Biol.

[7] Hillis D, Heath T, St John K (2005). Analysis and visualization of tree space. Syst Biol.

[8] Huson DH, Rupp R, Scornavacca C (2011). Phylogenetic Networks: Concepts, Algorithms and Applications.

[9] Steel M, Warnow T (1993). Kaikoura tree theorems: computing the maximum agreement subtree. Inf Process Lett.

[10] Stockham C, Wang LS, Warnow T. Statistically based prostprocessing of phylogenetic analysis by clustering. Bioinformatics. 2002;18:S285-S293. 10.1093/bioinformatics/18.suppl_1.s285 12169558

[11] Amenta N, Klingner J (2002). IEEE Symposium on Information Visualization. Case study: visualizing sets of evolutionary trees.

[12] Lee J, Verleysen M (2007). Nonlinear Dimensionality Reduction.

[13] Setiamarga D, Miya M, Yamanoue Y, Mabuchi K, Satoh T, Inoue J, Nishida M (2008). Interrelationships of atherinomorpha (medakas, flyingfishes, killifishes, silversides, and their relatives): the first evidence based on whole mitogenome sequences. Mol Phylogenet Evol.

[14] Kjer KM, Honeycutt RL (2007). Site specific rates of mitochondrial genomes and the phylogeny of eutheria. BMC Evol Biol.

[15] Zhang P, Papenfuss T, Wake M, Qu L, Wake D (2008). Phylogeny and biogeography of the family Salamandridae (Amphibia: Caudata) inferred from complete mitochondrial genomes. Mol Phylogenet Evol.

[16] Swofford DL. PAUP*: phylogenetic analysis using parsimony (* and other methods). 2002, Version 4:B10.

[17] Felsenstein J (1985). Confidence limits on phylogenies: an approach using the bootstrap. Evolution.

[18] Yang Z (1994). Estimating the pattern of nucleotide substitution. J Mol Evol.

[19] Yang Z (1994). Maximum likelihood phylogenetic estimation from DNA sequences with variable rates over sites: Approximate methods. J Mol Evol.

[20] Wilgenbusch JC. *Repmaker*. https://github.com/jwilgenb/repmaker/releases/tag/v1.0.1. 2016.

[21] Robinson D, Foulds L (1979). Comparison of weighted labelled trees. Lect notes Math.

[22] Robinson DF, Foulds LR (1981). Comparison of phylogenetic trees. Math Biosci.

[23] Owen M, Provan JS (2011). A fast algorithm for computing geodesic distances in tree space. IEEE/ACM Trans Comput Biol Bioinforma.

[24] Huang W, Zhou G, Marchand M, Ash JR, Morris D, Van Dooren P, Brown JM, Gallivan KA, Wilgenbusch JC. TreeScaper: visualizing and extracting phylogenetic signal from sets of trees. Mol Biol Evol. 2016;33:3314–16. 10.1093/molbev/msw196 27634869

[25] Grassberger P, Procaccia I (1983). Measuring the strangeness of strange attractors. Phys D Nonlinear Phenom.

[26] Camastra F, Vinciarelli A (2002). Estimating the intrinsic dimension of data with a fractal-based method. IEEE Trans Pattern Anal Mach Intell.

[27] Levina E, Bickel P. Maximum likelihood estimation of intrinsic dimension. In Advances in Neural Information Processing Systems. Volume 48109. Volume 17. Cambridge, MA, USA: The MIT Press; 2004.

[28] Pettis KW, Bailey TA, Jain AK, Dubes RC (1979). An intrinsic dimensionality estimator from near-neighbor information. IEEE Trans Pattern Anal Mach Intell.

[29] Borg I, Groenen PF (2005). Modern Multidimensional Scaling: Theory and Applications.

[30] Kruskal JB (1964). Multidimensional scaling by optimizing goodness of fit to a nonmetric hypothesis. Psychometrika.

[31] Sammon JW (1969). A nonlinear mapping for data structure analysis. IEEE Trans Comput.

[32] Demartines P, Herault J (1997). Curvilinear component analysis: a self-organizing neural network for nonlinear mapping of data sets. IEEE Trans Neural Netw.

[33] Van der Maaten LJP, Postma EO, Van Den Herik HJ (2009). Dimensionality reduction: a comparative review. J Mach Learn Res.

[34] Kaski S, Nikkila J, Oja M, Venna J, Toronen P, Castren E (2003). Trustworthiness and metrics in visualizing similarity of gene expression. BMC Bioinformatics.

[35] Wortley AH, Rudall PJ, Harris DJ, Scotland RW (2005). How much data are needed to resolve a difficult phylogeny? case study in lamiales. Syst Biol.

[36] Lecointre G, Philippe H, Vân Lê HL, Le Guyader H (1994). How many nucleotides are required to resolve a phylogenetic problem? The use of a new statistical method applicable to available sequences. Mol Phylogenet Evol.

[37] Felsenstein J, Kishino H (1993). Is there something wrong with the bootstrap on phylogenies? A reply to Hillis and Bull. Syst Biol.

[38] Qiu X, Fox GC, Yuan H, Bae SH, Chrysanthakopoulos G, Nielsen HF. Parallel Clustering and Dimensional Scaling on Multicore Systems. Springer Berlin/Heidelberg LNCS; 2008.

[39] Waterman MS, Smith TF (1978). On the similarity of dendrograms. J Theor Biol.

[40] Estabrook GF, McMorris FR, Meacham CA (1985). Comparison of undirected phylogenetic trees based on subtrees of four evolutionary units. Syst Biol.

[41] Heinsen A, Bendtsen F, Fomsgaard A (2000). A phylogenetic analysis elucidating a case of patient-to-patient transmission of hepatitis C virus during surgery. J Hosp Infect.

[42] Kuhner MK (2006). LAMARC 2.0: maximum likelihood and Bayesian estimation of population parameters. Bioinformatics.

[43] Billera LJ, Holmes SP, Vogtmann K (2001). Geometry of the space of phylogenetic trees. Adv Appl Math.

[44] Bogdanowicz D (2008). Comparing phylogenetic trees using a minimum weight perfect matching. Proc Int Conf Inf Technol.

[45] Robert CP, Casella G. Monte Carlo Statistical Methods. New York, New York, USA: Spring; 1999.

[46] Pesin YB. On rigorous mathematical definition of the correlation dimension and generalized spectrum fordimension. J Stat Phus. 1993;71:529–47.

[47] Snyder DL (1975). Random Point Processes.

[48] De Leeuw J (1988). Convergence of the majorization method for multidimensional scaling. J Classif.

[49] Ortega JM, Rheinboldt WC (1970). Iterative solution of nonlinear equations in several variables.

