# Supplementary material for: Visualizing phylogenetic tree landscapes
Source: BMC Bioinformatics. 2017 Feb 2;18:85. doi: 10.1186/s12859-017-1479-1 (PMC5290614; doi:10.1186/s12859-017-1479-1)
Supplement: Additional file 1: — Appendix. Detailed description of the intrinsic dimensionality measures, nonlinear dimensionality reduction methods, and the goodness of fit measures [45–49]. (PDF 195 kb) [file 12859_2017_1479_MOESM1_ESM.pdf]

Appendix

This appendix provides details of the methods used for intrinsic dimensionality estimation, nonlinear dimensionality reduction, and comparing the effectiveness of the dimension reduction methods.

Intrinsic Dimensionality Measures

Correlation Dimension

Let μ be a Borel probability measure on a metric space $\mathbb{Y}$ and for $q \geq 0$ and $\epsilon > 0$ define the quantity

$$C_q(\mu, \epsilon) = \int [\mu(\bar{B}_\epsilon(y))]^{q-1} d\mu(y) \tag{1}$$

where $\bar{B}_\epsilon(y)$ is the closed ball of radius ϵ centered at y. For $q \geq 0, q \neq 1$, the lower and upper q-dimensions of μ are defined as:

$$D_q^-(\mu) = \liminf_{\epsilon \to 0} \frac{\log C_q(\mu, \epsilon)}{(q-1)\log \epsilon} \tag{2}$$

$$D_q^+(\mu) = \limsup_{\epsilon \to 0} \frac{\log C_q(\mu, \epsilon)}{(q-1)\log \epsilon}. \tag{3}$$

When the lower and upper dimensions are equal, their value, $D_q(\mu)$, is the q-dimension of μ [46]. The correlation dimension is the special case of $q = 2$ [25].

If this dimension estimator is applied to a countable set of points, $Y = \{y_1, y_2, \cdots\}$, (1) becomes

$$\begin{aligned} C_2(\epsilon) &= \lim_{N \to \infty} \frac{1}{N(N-1)} \sum_{i=1, i<j}^{N} H(\epsilon - \|y_i - y_j\|_2) \\ &= P(\|y_i - y_j\|_2 \leq \epsilon), \end{aligned}$$

where $H(x) = 0$ if $x < 0$ and $H(x) = 1$ if $x \geq 0$. When it exists the correlation dimension has the form

$$d_{COR} = D_2(\mu) = \lim_{\epsilon \to 0} \frac{\log C_2(\epsilon)}{\log \epsilon}.$$

Maximum Likelihood estimator

This estimator assumes that the points are $Y = \{y_1, y_2, \cdots, y_N\}$ independently identically distributed samples from some probability density $f(y)$. $f(y)$ is assumed to be approximately constant near any point y_i. It is also assumed that the number of points in $\bar{B}_t(y)$ follow a inhomogeneous spatial Poisson process, $P(t, y)$ with rate

$$\lambda(t) = f(y)V(d_{ML})d_{ML}t^{d_{ML}-1}$$

where

$$V(d_{ML}) = \pi^{d_{ML}/2}[\Gamma(d_{ML}/2 + 1)]^{-1}$$

is the volume of the unit sphere in $\mathbb{R}^{d_{ML}}$ and $\Gamma(z)$ is the Gamma function. The log-likelihood of the process $P(t, y)$ is

$$L(d_{ML}, \theta) = \int_0^R \log \lambda(t) dP(t, y) - \int_0^R \lambda(t) dt,$$

where $\theta = \log f(y)$ and R is the radius of the ball around y on which f is approximately constant [47]. The dimension, as a function of R and y, at which $L(d_{ML}, \theta)$ is maximized is

$$\hat{d}_{ML}(R, y) = \left\{ \frac{1}{P(R, y)} \sum_{j=1}^{P(R,y)} \log \frac{R}{T_j(y)} \right\}^{-1} \tag{4}$$

Levina and Bickel [27] suggest that, in practice, it is more convenient to fix the number of neighbors, k, rather than the radius and replace (4) with

$$\hat{d}_{ML}(k, y) = [\frac{1}{k-1} \sum_{j=1}^{k-1} \log \frac{T_k(y)}{T_j(y)}]^{-1},$$

where $T_k(y)$ is the distance between point y and k-th nearest point to y. Rather than choosing a single y_i from the set, the mean

$$\bar{d}_{ML}(k) = \frac{1}{N} \sum_{i=1}^{n} \hat{d}_{ML}(k, y_i).$$

is considered for a range of k values. The interval $[k_1, k_2]$ is chosen so that the profile of $\bar{d}_{ML}(k)$ is close to flat and the estimate of the dimension is defined as

$$d_{ML} = \frac{1}{k_2 - k_1 + 1} \sum_{k=k_1}^{k_2} \bar{d}_{ML}(k).$$

Nearest Neighbor estimator

This estimator assumes that the points are $Y = \{y_1, y_2, \cdots, y_N\}$ independently identically distributed samples from some probability density $f(y)$. Pettis et al. [28] derive the approximation

$$f(y) \approx \hat{f}(y) = \frac{k}{NV(d_{NN})} [T_k(y)]^{-d_{NN}}$$

where $V(d)$ and $T_k(y)$ are defined as in the Maximum Likelihood estimator. This yields

$$\log k = d_{NN} \log \bar{T}_k(y) + d_{NN} \log(G_{k,d_{NN}}) - d_{NN} \log(C_n),$$

where $\bar{T}_k(y) = (1/N) \sum_{i=1}^{N} T_i(y)$, $G_{k,d_{NN}} = k^{1/d} \Gamma(k) / \Gamma(k + 1/d)$ and $C_N = (1/N) \sum_{i=1}^{N} [N f(y_i) V_{d_{NN}}]^{-1/d}$. Pettis et al. [28] show that C_n is independent of

k and $\log(G_{k,d})$ is close to 0. So the relation between $\log k$ and $\log \bar{T}_k(y)$ is close to a linear function with slope d_{NN}.

A nearest neighbor estimator can therefore be computed using the algorithm:
1. compute $\bar{T}_k = \frac{1}{N}\sum_{i=1}^{N} T_k(y_i)$, for $k = 1, 2, \cdots$
2. plot the figure with $\log k$ versus $\bar{T}_k$
3. estimate the slope, and the slope is d_{NN}

Visual Inspection

Lee and Verleysen [12] suggested estimating the dimension by examining the profile of the final value of the NLDR cost function as a function of the dimension to which the data is reduced. The dimension estimate is taken to be an integer value in the interval where the profile is relatively flat.

Nonlinear Dimensionality Reduction Methods

In this section, we present the basic definitions of the stress functions and optimization algorithms. Difficulties or important optimizations for particular pairs are discussed briefly.

In the discussions below, we assume that we are given N points, $Y = \{y_1, y_2, \cdots, y_N\}$, in a space, $\mathcal{S}$, not necessarily Euclidean, and an associated distance function, $dist(y_i, y_j)$. NLDR produces N points in $\mathbb{R}^s$, $\mathcal{X} = \{\mathbf{x}_1, \mathbf{x}_2, \cdots, \mathbf{x}_N\}$ that are represented by the $n = Ns$ components of $X = [x_1, x_2, \cdots, x_n]$. We assume that successive groups of s components of X define projected vector $\mathbf{x}_i$, i.e., $X = vec(\mathbf{x}_1, \mathbf{x}_2, \cdots, \mathbf{x}_N)$. In the following formulas, $\hat{d}_{ij} = dist(y_i, y_j)$ and $d_{ij}(X) = \|\mathbf{x}_i - \mathbf{x}_j\|$ for some norm on $\mathbb{R}^s$.

Stress Functions

We consider four stress functions: Normalized stress [29], Kruskal-1 stress [30], Sammon's stress, also known as the nonlinear mapping (NLM) stress [31], and Curvilinear Components Analysis (CCA) stress [32].

The Normalized stress function is defined as

$$\sigma_n(X) = \frac{\sum_{i<j}(d_{ij}(X) - \hat{d}_{ij})^2}{\sum_{i<j}\hat{d}_{ij}^2}.$$

This stress function is normalized by a single weight that is related to the data in $\mathcal{S}$ and is therefore a constant, i.e., independent of the projection onto $\mathbb{R}^s$.

The Kruskal-1 stress function is defined as

$$\sigma_1^2(X) = \frac{\sum_{i<j}(d_{ij}(X) - \hat{d}_{ij})^2}{\sum_{i<j} d_{ij}^2(X)}.$$

It is normalized by a single weight but this weight is determined by the projected data in $\mathbb{R}^s$. However, the minimizers of Kruskal-1 stress function are the same as Normalized Stress function after a rescaling of X, i.e., $\sigma_n(X) = \sigma_1^2(\gamma(X)X)$ where $\gamma(X) \in \mathbb{R}$.

Sammon's stress function or the NLM stress function is defined as

$$\sigma_s = \frac{1}{c}\sum_{i<j}\frac{(d_{ij}(X) - \hat{d}_{ij})^2}{\hat{d}_{ij}},$$

where $c = \sum_{i<j}\hat{d}_{ij}$. Note that this stress function has a weight, $dist(y_i, y_j)$, for each term in the sum of squares of the distance differences. Sammon's stress function aims to preserve distances between points that are close in $\mathcal{S}$ when assigning positions in $\mathbb{R}^s$.

Like Sammon's stress function, the CCA stress function also has local weights. However, CCA's weights are related to the points in $\mathbb{R}^s$. The CCA stress function is defined as

$$\sigma_c = \frac{1}{2}\sum_{ij}(d_{ij}(X) - \hat{d}_{ij})^2 H_\lambda(d_{ij}(X)),$$

where the weight function $H_\lambda(z)$ is a decreasing positive function dependent on a parameter λ. Demartines and Herault [32] discuss two choices for this function. They are $H_\lambda(z) = exp(-z/\lambda)$ and $H_\lambda(z) = 1_{(\lambda-z)}$, where $1_{(u)} = 1$, if $u > 0$ and $1_{(u)} = 0$, if $u <= 0$. The value of λ decreases on each iteration. Initially, it is large and the function H_λ is close to 1 and $\sigma_c(X)$, like $\sigma_n(X)$, considers all points similarly. As the iteration proceeds, λ decreases and the importance of a term in the stress function depends on the proximity of the associated pair of points in $\mathbb{R}^s$. Therefore, the number of points involved in updating the positions in X generally decreases as the iteration progresses.

The manner in which λ is updated is very important to the applicability of this cost function and depends on the algorithm used to optimize $\sigma_{cca}(X)$. In particular, the influence of the update of λ on the relationship of the convergence behavior and the complexity of an iteration of the optimization algorithm is a crucial consideration. The details of this consideration for each algorithm below are omitted but the interested reader is directed to [Section 4.2.4, 12] for a discussion of this issue for simple gradient descent and the Stochastic Gradient Descent algorithms.

Optimization Algorithms

We have considered four algorithms to optimize the stress functions. They are majorization , Gauss-Seidel-Newton, stochastic gradient descent, and MCMC simulated annealing. The first two are classical descent methods while the latter two are based on stochastic ideas that allow occasional ascent steps.

Majorization

The majorization algorithm is a classical approach to minimizing a function $F(X)$ that has been applied in the NLDR context by [48]. A function $G(X, Z)$ which satisfies $G(X, X) = F(X)$ and $G(X, X) < G(X, Z)$ is called a majorizing function. Minimizing $F(X)$ directly means searching for minimum along line $Z = X$. The majorization algorithm exploits Z to search along another path. Given a majorizing function, we have

$$F(X) = G(X, X) \leq G(X, Z).$$

and the algorithm is
0. Given $Z_0 = X_0$, k = 1
1. $X_k = \text{argmin}_X G(X, Z_{k-1})$
2. $Z_k = X_k$
3. if stopping condition not satisfied then $k = k + 1$ and go to step 1 .

According to the properties of majorizing function and taking X_k as a minimizer we have

$$\begin{aligned} F(X_k) &= G(X_k, X_k) \\ &\leq G(X_k, X_{k-1}) \\ &\leq G(X_{k-1}, X_{k-1}) \\ &\leq F(X_{k-1}) \end{aligned}$$

and a sequence X_i such that $F(X_i)$ is a nonincreasing sequence is produced. This method usually converges to a local minimum. The main limitation of majorization is, of course, the need for a majorizing function. However, even when $G(X, Z)$ exists, finding the minimizer over X may be more difficult than the original problem. The lack of a suitable majorizing function is the reason, in practice, why majorization is not used for NLDR based on $\sigma_{cca}(X)$. For $\sigma_n(X)$, $\sigma_1(X)$, and $\sigma_s(X)$, analytical expressions for the minimizer of $G(X, Z)$ over x given a value of Z are known for the respective majorizing functions.

Gauss-Seidel-Newton

Coordinate descent methods are another classical approach to minimization that adapts standard methods for solving nonlinear equations to optimization [49]. Their advantage is their simplicity and while their convergence, in general, may be unacceptably slow they can be effective for particular problems.

The basic idea is to find a root of the gradient of $F(X)$, i.e., $\nabla F(X^*) = 0$ by allowing only a subset of the variables to change. The simplest approach is to fix all components of X except one that is updated in a manner that reduces $F(X)$ and moves towards a root of $\nabla F(X)$. Each of the components of X are updated based on some schedule that defines the method and each update requires taking one of more steps of a scalar nonlinear equation solver such as Newton's method. The one-step Gauss-Seidel-Newton method is so-called because a Gauss-Seidel relaxation schedule is used to order the updates of the components of X, i.e., when updating a component of X the most recent value of all other components are used to compute a single scalar Newton's method update. Specifically, we have

$$\nabla F(X) = \begin{pmatrix} \nabla_1 F(x_1, x_2, ..., x_n) \\ \nabla_2 F(x_1, x_2, ..., x_n) \\ ... \\ \nabla_n F(x_1, x_2, ..., x_n) \end{pmatrix}$$

and the one-step nonlinear Gauss-Seidel-Newton algorithm updates the scalar $x_i^{(k)}$ with $\Delta_i^{(k+1)} = x_i^{(k+1)} - x_i^{(k)}$ where

$$\Delta_i^{(k+1)} = -\alpha \frac{\nabla_i F(x_1^{(k+1)}, x_2^{(k+1)}, ..., x_{i-1}^{(k+1)}, x_i^{(k)}, x_{i+1}^{(k)}, \ldots, x_n^{(k)})}{|\nabla_i^2 F(x_1^{(k+1)}, x_2^{(k+1)}, ..., x_{i-1}^{(k+1)}, x_i^{(k)}, x_{i+1}^{(k)}, \ldots, x_n^{(k)})|},$$

$1 \leq i \leq n$, $k = 0, 1, ...$ where $\alpha > 0$ is stepsize. Note the algorithm takes a single step of scalar Newton's method to update each component of X. The absolute value guarantees that the method is a descent method for infinitesimal α. Sammon applied one-step Jacobi-Newton to the NLDR problem using $\sigma_s(X)$, i.e., each component was updated using the old values of all other components, and a fixed stepsize but did nothing to guarantee a descent step [31]. The one-step Jacobi-Newton method can also be viewed as an inexact Newton method to update X where the Hessian is approximated by its diagonal elements only. In order to guarantee a descent step with a nontrivial α we use a line search and impose the strong Wolfe conditions to determine α on each step. Note that the algorithm is easily extended to the block case where, rather than updating a single component of X, the s components that define the projected vector $\mathbf{x}_i$ are updated using one step of Newton's method for a system of s nonlinear equations. In that case, the notion of the absolute value is, of course, no longer a valid method of guaranteeing descent for the $s \times s$ local Hessians.

One-step Gauss-Seidel-Newton is easily applied to $\sigma_n(X)$, $\sigma_1(X)$, and $\sigma_s(X)$. For $\sigma_{cca}(X)$ the update of λ and its relationship to convergence yields a noticeable increase in the complexity of a single iteration compared to the other algorithms applied to $\sigma_{cca}(X)$.

Stochastic Gradient Descent

The majorization and Gauss-Seidel-Newton algorithms discussed above are classical descent techniques. The remaining two algorithms we have considered have a statistical approach that allows the occasional ascent step, particularly early in the iteration, in order to provide a more global view of the cost function and convergence. The Stochastic Gradient Descent method (SGD) modifies the classical deterministic descent method, see [Appendix C, 12] for a description. Note that the uses of the name Stochastic Gradient vary considerably in the numerical literature. We adopt it here due to its use for this algorithm for NLDR by [12]. It is used also to describe algorithms based on various forms of probabilistic perturbations to a deterministic gradient used for example in the MCMC Simulated Annealing algorithm discussed in the next section.

It is assumed that the cost function $F(X)$ has the form

$$F(X) = \sum_{i=1}^{N} g_i(X)$$

where each term $g_i(X)$ is associated with the distances from one of the projected vectors, $\mathbf{x}_i$, to all of the others. The deterministic gradient descent algorithm up-

dates the s components of X corresponding to each projected vector $\mathbf{x}_j \in \mathbb{R}^s$ using

$$\mathbf{x}_j^{(k+1)} \leftarrow \mathbf{x}_j^{(k)} - \alpha \nabla_{\mathbf{x}_j} \sum_{i=1}^{N} g_i(X) = \mathbf{x}_j^{(k)} - \alpha \sum_{i=1}^{N} \nabla_{\mathbf{x}_j} g_i(X).$$

where $\alpha > 0$ is set based on optimization considerations. SGD updates each component of X using, $g_i(X)$, a single randomly selected term in the $F(X)$. Given the choice of i, the update is

$$\mathbf{x}_j^{(k+1)} \leftarrow \mathbf{x}_j^{(k)} - \alpha \nabla_{\mathbf{x}_j} g_i(X)$$

for $j = 1, 2, \cdots, n$. To ensure convergence, the step size α is set based on a Robbins-Monro condition [12].

Demartines and Herault [32] applied a version of SGD to $\sigma_{cca}(X)$. It is easily applied to the other cost functions since all can be written in the assumed form of $F(X)$. When applied to $\sigma_n(X)$, $\sigma_s(X)$, and $\sigma_{cca}(X)$, $\nabla_{\mathbf{x}_j} g_i(X)$ depends only on $\mathbf{x}_j$ and $\mathbf{x}_i$. However, when applied to $\sigma_1(X)$ the update of each $\mathbf{x}_j$ is significantly more complex, by a factor of $O(N)$, than the updates for the This is due to the dependence on all of the $\mathbf{x}_i$ in the weight $(\sum_{i<j} d_{ij}^2(X))^{-1}$.

MCMC Simulated Annealing algorithm

The final optimization algorithm used is the MCMC Simulated Annealing algorithm which is discussed in detail in [45]. The method combines a discrete MCMC iteration that samples a distribution related to a cost function $F(X)$ and determinstic simulated annealing to transform the sampling into an efficient optimization algorithm for $F(X)$. The natural form of the algorithm to find a maximum of a function $F(X)$ is used here. It is easily modified for minimization by applying it to $-F(X)$.

The MCMC aspect of the algorithm is derived by combining a diffusion process with a Metropolis-Hastings-type acceptance-rejection test. Consider the probability density $p(X) = e^{F(X)}/G$, where G is a normalized constant which makes the integration of $p(X)$ equal to 1. Note that the peaks of $p(X)$ occur at local maxima of $F(X)$. Therefore, a brute force method of determining the location of a maximum is to repeatedly sample X using the density $p(X)$. After a large number of samples, the frequency of the X observed would indicate a peak in the density and a maximum of $F(X)$. The sampling is accomplished using a proposal density $q(Z|X)$ and the acceptance-rejection function $\rho(X, Z) = \min\{\frac{p(Z)q(X|Z)}{p(X)q(Z|X)}, 1\}$. For MCMC Simulated Annealing, the proposal density $q(Z|X)$ is taken as the normal density with mean $X + \nabla F(X)\delta$ and variance δ. This implies that the acceptance-rejection function is $\rho(X, Z) = e^{F(Z)-F(X)}$. Note that repeatedly sampling from the proposal density function, i.e., $X_{t+1} \sim q(Z|X_t)$, corresponds to following the deterministic gradient with a random perturbation on each step. This is a diffusion process also referred to as a stochastic gradient flow. The addition of the Metropolis-Hastings acceptance-rejection step yields a discrete MCMC algorithm, the Metropolis-adjusted Langevin algorithm, that samples $p(X)$.

This sampling algorithm is a very inefficient method of optimization. This is fixed by applying simulated annealing and adding a temperature T to the density

function $p(X)$ that becomes $p_T(X) = \frac{e^{F(X)/T}}{G_T}$. When T is large, p_T is close to a uniform distribution and when T is small, p_T has sharp peaks. Starting with a large T and reducing it according to a certain schedule yields a globally convergent maximization algorithm. When T is large, the samples are generated from an almost uniform distribution, i.e, the samples are free to roam globally. As T decreases, the samples range reduces to concentrate on neighborhoods of peaks and eventually converging to one of them.

The resulting algorithm that can be efficiently applied to all of the NLDR cost functions described earlier is given by
MCMC Simulated Annealing algorithm:
0. Initialize $T > 0$ and large. Initialize $0 < c < 1$ and X_1. Let $t = 1$;
1. Generate a candidate $Z \sim N(x + \nabla F(x)\delta, \delta T)$.
2. Update the iterate to X_{t+1} according to:

$$X_{t+1} = \begin{cases} Z, & \text{with probability } \rho(X_t, Z); \\ X_t, & \text{with probability } 1 - \rho(X_t, Z). \end{cases}$$

where $\rho(X, Z) = \min\{e^{(F(Z)-F(X))/T}, 1\}$.
3. update $T = cT$, $t = t + 1$, go to Step 1. (We have used $c = 0.96$.)

Goodness of Fit

The measures of goodness of fit used in this study are 1 Nearest Neighbor (1NN) [33], Continuity [34], and Trustworthiness [34].

1NN assumes that the original data $Y = \{y_1, y_2, \cdots, y_N\}$ in $\mathcal{S}$ have been assigned to clusters or classes. The projected Euclidean representations $\mathcal{X} = \{\mathbf{x}_1, \mathbf{x}_2, \cdots, \mathbf{x}_N\}$ inherit the cluster/class of the corresponding y_i. 1NN computes the percentage of the points whose nearest neighbors in the projected space $\mathbb{R}^s$ belong to a same cluster. This can be computed by:
0. $k = 1$; $T = 0$;
1. compute $\mathbf{x}_{i_k}$ which is closest to $\mathbf{x}_k$.
2. if y_{i_k} and y_k belong to a same cluster, then $T = T + 1$;
3. $k = k + 1$. if $k <= n$, then goto step 1.
4. output T/n
Note that the measure is simple to extend to examine the r closest neighbors of $\mathbf{x}_k$.

Continuity is based on the notion that if the two points $y_i,\ y_j \in \mathcal{S}$ are close to each other measured by $dist(y_i, y_j)$, the corresponding points $\mathbf{x}_i,\ \mathbf{x}_j \in \mathbb{R}^s$ should be close to each other measured by $\|\mathbf{x}_i - \mathbf{x}_j\|$. It punishes the points that are in the neighborhood of $y_i \in \mathcal{S}$ but not in the neighborhood $\mathbf{x}_i \in \mathbb{R}^s$.

Given a positive integer k, the neighborhood of point with index i comprises the k-nearest points. Let $V_k(i)$ denote the set of indices of points that are in the neighborhood of size k of the point $y_i \in \mathcal{S}$ but are not in the neighborhood of size k of the point $\mathbf{x}_i \in \mathbb{R}^s$. Also let $s(i, j)$ represent the rank of the $\mathbf{x}_j$ in the list of the elements of $\mathcal{X}$ ordered by distance from $\mathbf{x}_i$. The measure of continuity is then given by

$$Con = 1 - \frac{2}{nk(2n - 3k - 1)} \sum_{i=1}^{n} \sum_{j \in V_k(i)} (s(i, j) - k). \tag{5}$$

The weight is the worst case value of the sum and therefore normalizes Con.

Trustworthiness reverses the roles of $\mathcal{S}$ and $\mathbb{R}^s$ as used in Continuity. It punishes the points that are in the neighborhood $\mathbf{x}_i \in \mathbb{R}^s$ but not in the neighborhood of $y_i \in \mathcal{S}$. Let $U_k(i)$ denote the set of indices of points that are in the neighborhood of size k of the point $\mathbf{x}_i \in \mathbb{R}^s$ but are not in the neighborhood of size k of the point $y_i \in \mathcal{S}$. Also let $r(i,j)$ represent the rank of the y_j in the list of the elements of Y ordered by distance from y_i. The measure of trustworthiness is then given by

$$Tru = 1 - \frac{2}{nk(2n-3k-1)} \sum_{i=1}^{n} \sum_{j \in U_k(i)} (r(i,j) - k). \tag{6}$$

The weight is the worst case value of the sum and therefore normalizes Tru.
